# Lessons from a phenotypically normal infant with uniparental isodisomy of chromosome 21: a Case Report and review

**DOI:** 10.3389/fgene.2025.1544565

**Published:** 2025-03-05

**Authors:** Yuying Zhu, Ke Wu, Cuicui Jiang, Qiumin Zhu

**Affiliations:** ^1^ Prenatal Diagnosis Center, Quzhou Maternal and Child Healthcare Hospital, Quzhou, Zhejiang, China; ^2^ Laboratory of Prenatal Diagnosis Center, Quzhou Maternal and Child Healthcare Hospital, Quzhou, Zhejiang, China; ^3^ Obstetrics Department, Quzhou Maternal and Child Healthcare Hospital, Quzhou, Zhejiang, China

**Keywords:** uniparental isodisomy, region of homozygosity, whole-exome sequencing, genetic counseling, mosaicism

## Abstract

Uniparental disomy (UPD) occurs when both homologous chromosomes are inherited from a single parent. To date, the UPD of all autosomes and the X chromosome has been recorded. A few cases of UPD of chromosome 21 have been documented. At 15 weeks of gestation, a 25-year-old pregnant woman’s non-invasive prenatal screening revealed a high risk of trisomy 21. Although no anomalies were detected in the fetal ultrasonography, amniocentesis was performed, and the fetal karyotype analysis was found normal. A single-nucleotide polymorphism (SNP) array revealed that the fetus had the copy-neutral region of homozygosity (ROH) in the long arm of chromosome 21. Subsequently, single whole-exome sequencing was performed due to the risk of recessive gene variants in ROH, and no homozygous like pathogenic or pathogenic variants were found on the long arm of chromosome 21. After genetic counseling, the parents decided to continue this pregnancy. At 37 weeks of gestation, a live male infant was delivered by Cesarean section. Copy number variation sequencing showed that the placental tissue was mosaic for trisomy 21. At the final follow-up evaluation, the 6-month-old boy had a normal phenotype.

## Introduction

In uniparental disomy (UPD), both homologous chromosomes are inherited from a single parent. Based on whether both homologous chromosomes from one parent are identical, there are two subtypes of UPD: uniparental heterodisomy (UPhD) and uniparental isodisomy (UPiD). Mechanisms leading to UPD include trisomic/monosomic rescue, gamete complementation, and postfertilization errors (Liehr, 2022). Genomic imprinting depends on the parental origin of the imprinted genes, thereby resulting in the non-equivalent expression of maternal and paternal genomes (Eggermann, 2024). UPD could lead to imprinting disorders. To date, the UPD of all autosomes and the X chromosome has already been recorded. Studies have reported UPiD-caused autosomal recessive diseases detected by whole-exome sequencing. Few cases of UPD of chromosome 21 have been documented. Herein, we report a phenotypically normal infant with UPiD (21), explore previously published cases, and aim to provide useful lessons for clinical diagnosis in the future.

## Materials and methods

A 25-year-old pregnant woman (gravida 0, para 0) was referred to the Center of Prenatal Diagnosis at Quzhou Maternal and Children Hospital for genetic counseling. At 15 weeks of gestation, the pregnant woman’s non-invasive prenatal screening (NIPS) showed a high risk of trisomy 21 (Z-score, 6). The patient signed an informed consent for her genetic analysis and amniocentesis. The fetal ultrasonography indicated no anomalies before the amniocentesis. Subsequently, the amniocentesis was performed at 18 weeks of gestation, and the fetal sample was detected by single-nucleotide polymorphism (SNP) array analysis, and G-banding karyotype analysis with the 400-band level.

### Genetic tests of the placenta sampling

To examine the reason behind the false positive of NIPS, copy number variation sequencing (CNV-seq) was performed with low read-depth (3×) on placental tissues, umbilical cord, and cord blood for detecting the ploidy (number of sets of chromosomes in a cell or organism). Soybean-sized placental tissues symmetrically positioned at specific depths were obtained from the fetal and maternal sides of the placenta, respectively. Six samples were collected: two from the maternal side of the center of the placenta, two from the fetal side of the edge of the placenta, one umbilical cord, and one cord blood sample**.**

## Results

### G-banding and C-banding karyotype analysis

The G-banding karyotype analysis of 30 metaphases revealed a normal fetal amniotic fluid.

### Chromosomal microarray analysis

**FIGURE 1:**
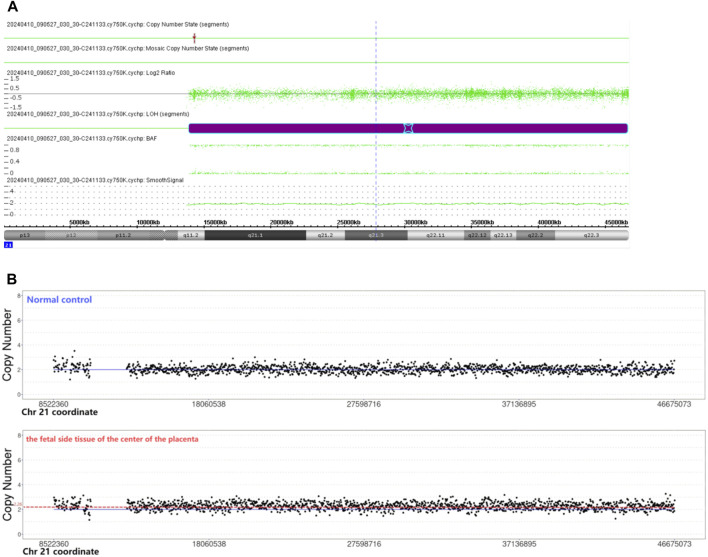
**(A)** Chromosomal microarray analysis indicated the copy-neutral region of homozygosity (ROH) in the long arm of chromosome 21. Smooth signal representing a normal copy number (green line) along the arm of chromosome 21. The B Allele Frequency (BAF) representing AA, and BB alleles (two green lines). **(B)** CNV-seq revealed the copy number of the fetal side tissue on the center of the placenta was 2.26. The copy number of the normal control was 2.

The chromosomal microarray analysis (CMA) was done using an SNP array (Affymetrix CytoScan 750K Array, Santa Clara, California). It revealed that the fetus had the copy-neutral region of homozygosity (ROH) in the long arm of chromosome 21 (Figure 1A).

### Whole-exome sequencing

Due to the risk of recessive gene variants in ROH, single WES was recommended, and WES found no homozygous likely pathogenic or pathogenic variants on the long arm of chromosome 21.

### Pregnancy outcome

The pregnant woman was informed of these genetic results. There were no abnormal findings on the ultrasound throughout the entire pregnancy. After genetic counseling, this family decided to continue the pregnancy of the women. At 37 weeks of gestation, a live male infant was delivered by Cesarean section, with a length of 50 cm and a weight of 3,250 g. The 1-min and 5-min Apgar score were all 10. At the final follow-up evaluation, the 6-month-old male newborn demonstrated a normal phenotype.

### Genetic results of the placenta sampling

CNV-seq revealed mosaic trisomy 21 in only the fetal side tissue on the center of the placenta, the percentage of trisomy 21 mosaicism was about 26% (Figure 1B). The other five samples were all euploid.

## Discussion

Two copies of a single chromosome or chromosome segment are inherited from one parent, and no copy is inherited from the other parent, which is called UPiD (21). It was concluded that the positive result of NIPS was caused by the fetal side of the placenta of mosaic trisomy 21. It was presumed that ROH in the long arm of chromosome 21 is caused by a postzygotic trisomy 21 self-rescue event, the two remaining chromosomal 21 copies originated from the same parent, thereby resulting in UPiD (21). One in four placental samples was mosaic for trisomy 21; it suggests that postzygotic trisomy 21 trophectoderm does not rescue completely like inner cell mass, and collecting more than one placental sample is important to explore the mechanism of UPD.

There are two imprinted genes (MIR125B2, DSCAM) and one predicted imprinted gene (*SIM2*) found on chromosomal 21 according to the Geneimprint database (http://www.geneimprint.com/). The *MIRN125B2* gene maps to chromosome 21q21.1. The paternal expression of *MIR125B2* is ubiquitous in human tissues (Sonkoly et al., 2007). Chou et al. (2023) demonstrated that *MIR125B2* was only imprinted in the human brain, and is associated with cognitive impairment and brain hypotrophy. Patients with Down syndrome (DS) displayed an increased level of miR-125b-2 (Farroni et al., 2018). The *DSCAM* gene which maps to chromosome 21q22.2-q22.3 is a paternally expressed imprinted gene in the human placenta, which would not be affected by the presence of the supernumerary chromosome 21 (Allach El Khattabi et al., 2019). *DSCAM* may be a candidate gene responsible for intellectual disability (Yamakawa et al., 1998), and cardiac and visceral malformations (Jannot et al., 2013).

To date, a few published cases of UPD(21) have been reported. We excluded UPD(21) cases with mosaic trisomy 21 (Bruyere et al., 2000; Chen et al., 2020; Chen et al., 2022; Chen et al., 2023), ring chromosome 21 (Bartsch et al., 1994) or a *de novo* mutation on the Y chromosome (Mansuet-Lupo et al., 2009). UPD(21) cases without available detailed clinical information were also excluded from the study (Nakka et al., 2019; Cavalheiro et al., 2020; Semikhodskii et al., 2023). So, only seven previously published cases of “pure” UPD(21) without mosaicism or other variations were assessed (Table 1).

**TABLE 1:** Clinical information on previously published cases of UPD(21) without mosaicism or other variations.

Cases	Results of molecular detection and chromosomal karyotype	Tested tissue	Clinical information
Créau-Goldberg et al. (1987)	mUPD(21) 45,XX,-21,-21,t (21; 21) (q10; q10), *de novo*	lymphocytes	The adult woman with normal phenotype had a newborn with trisomy 21 46,XY,t (21; 21) (q10; q10)
Blouin et al. (1993)	pUPiD (21), 45,XY,-21,-21,i (21q), *de novo*	lymphocytes	The 40-year-old man with normal phenotype had a trisomy 21 son
Robinson et al. (1994)	pUPiD (21), 45,XX,-21,-21,i (21q), *de novo*	NA	The adult woman with normal phenotype had a history of recurrent spontaneous abortion
Henderson et al. (1994)	mUPhD (21), 46,XX	product of conception	The mother was a 31 years old, the routine ultrasound at 8 weeks of gestation found no fetal pole
Rogan et al. (1999)	mUPhD (21) and mUPiD (21) were observed, 45,XY,-21,-21,der (21; 21) (q10; q10)	leukocytes	The one-year-old man was clinically and developmentally normal
Fritz et al. (2001)	pUPiD (21), 46,XX	product of conception (frozen fetal tissue)	The 40-year-old woman had spontaneous abortion, the focal hydrops and fibrosis of the placenta with chorionic villi showed decreased ramification and vascularization at 8 weeks of gestation
Pan et al. (2013)	UPiD (21), 46,XX	the amniotic fluid	The 42-year-old woman received an amniocentesis at 16 weeks of gestation because of the abnormal biochemical profile and the positive result of NIPS. No fetal structure anomaly was detected on the ultrasound examination, the couple decided to terminate the pregnancy
This case	UPiD (21), 46,XY, *de novo*	the amniotic fluid	The 6-month-old boy had normal phenotype

Two cases were products of conception with normal karyotypes. Four cases were postnatal cases with normal phenotypes and abnormal karyotypes. Though one case had a prenatal fetus with a normal karyotype, the couple decided to terminate the pregnancy.

We reported one case with UPiD (21) that is attributed to the mechanism of trisomic rescue, and reviewed previously published cases of UPD(21). Some findings from these cases are documented as under:

1) These two imprinted genes on chromosomal 21 might not be associated with abnormal phenotype or human disease, so the presence of UPD(21) in prenatal diagnosis would be considered a favorable outcome, thereby potentially influencing the decision regarding termination of pregnancy.

2) UPiD-caused autosomal recessive diseases detected by WES have been reported previously (Zhou et al., 2024; Lopez-Garrido et al., 2022). Although UPiD (21)-caused autosomal recessive diseases have not been reported, the utilization of WES is recommended for detecting homozygous likely pathogenic or pathogenic variants on chromosome 21.

3) If the NIPS suggests a high risk of trisomy 21, the presence of confined placental mosaicism (CPM) should be considered. However, CPM involving trisomy 21 has not shown an unfavorable effect on pregnancy outcomes (Thomsen et al., 2024; Grati et al., 2020).

4) The possibility of considering the chromosome-balanced translocation should be taken into account. UPD (21) can coexist with chromosome-balanced translocations, typically der (21; 21) (q10; q10). It is likely that these carriers may encounter recurrent spontaneous abortion and have a high risk of pregnancy with trisomy 21. Therefore, chromosomal karyotype analysis is also recommended.

Overall, we also describe a phenotypically normal 6-month-old boy with UPiD (21). We also review previously published cases and sum up some useful lessons for clinical diagnosis and prenatal diagnosis.

## Data Availability

Datasets are available on request: the raw data supporting the conclusions of this article will be made available by the authors, without undue reservation. Requests to access these datasets should be directed to [Qiumin Zhu, 946346439@qq.com].
